# Molecular Mechanisms Underlying the Anti-Tumor Activity of Lotus-Derived Alkaloids in Breast Cancer

**DOI:** 10.3390/molecules31060947

**Published:** 2026-03-12

**Authors:** Qinyi He, Ling Luo, Dezhao Zhang, Wenxiang Zhou, Ningning Bai, Canwei Du, Songlian Li

**Affiliations:** 1 National & Local Joint Engineering Laboratory of Animal Peptide Drug Development, Peptide and Small Molecule Drug R&D Platform of Furong Laboratory, College of Life Sciences, Hunan Normal University, Changsha 410081, China; 2 Hunan Engineering Research Center of Lotus Deep Processing and Nutritional Health Sciences, School of Life and Health Sciences, Hunan University of Science and Technology, Xiangtan 411201, China; 3 Department of Breast Surgery, Second Affiliated Hospital of Hunan University of Chinese Medicine, Changsha 410036, China

**Keywords:** breast cancer, liensinine, isoliensinine, neferine, MAPK signaling pathway, apoptosis, natural products

## Abstract

Breast cancer represents a persistent global health burden, marked by extensive molecular heterogeneity and frequent therapeutic resistance in aggressive subtypes, particularly triple-negative breast cancer (TNBC). These clinical challenges underscore the urgency for alternative therapeutic strategies. Bioactive alkaloids isolated from *Nelumbo nucifera*, especially the bisbenzylisoquinoline compounds liensinine (LIE), isoliensinine (ISO), and neferine (NEF), have emerged as promising candidates due to their ability to disrupt oncogenic signaling pathways and inhibit malignant cellular transformation. The present study conducted a systematic investigation of LIE, ISO, and NEF across multiple breast cancer cell lines, including highly aggressive TNBC models. Results revealed potent growth-inhibitory effects mediated through apoptosis induction and cell cycle arrest at both the G1 and G2/M phases. Furthermore, transcriptomic profiling and molecular analysis identified LIE as a principal effector, driving extensive transcriptional reprogramming and targeting the MAPK and mTOR pathways as core regulators of its anti-cancer efficacy. Collectively, these findings define a mechanistic framework for the anti-cancer potential of *N. nucifera*-derived alkaloids and provide a compelling foundation for their development as therapeutic candidates for advanced breast cancer.

## 1. Introduction

Breast cancer remains a leading global health concern, defined by malignant transformation and uncontrolled proliferation of mammary epithelial cells [1]. Predominantly affecting women, with male cases accounting for less than 1%, breast cancer is the fourth leading cause of cancer-related mortality globally, with 660,000 deaths reported in 2022 [2]. Pathogenesis typically initiates in the terminal duct lobular units and progresses through distinct histopathological stages, including ductal carcinoma in situ (DCIS), a localized non-invasive form, and invasive ductal carcinoma (IDC), characterized by stromal infiltration of malignant cells [3].

Molecular classification of breast cancer, based on immunohistochemical profiling of estrogen receptor (ER), progesterone receptor (PR), human epidermal growth factor receptor 2 (HER2), and Ki-67, delineates four principal subtypes: luminal A, luminal B, HER2-positive, and triple-negative breast cancer (TNBC) [4]. Luminal A tumors exhibit ER/PR positivity and low Ki-67 expression (<14%) and account for approximately 60–70% of cases, generally associated with favorable clinical outcomes. Luminal B tumors, comprising 10–20% of cases, display ER positivity alongside either HER2 positivity or elevated Ki-67 levels (≥14%) and often yield less favorable prognoses despite responsiveness to endocrine therapy. HER2-positive breast cancer, defined by ER/PR negativity and HER2 overexpression, represents 13–15% of cases and is managed through anti-HER2 agents combined with cytotoxic chemotherapy. TNBC, lacking expression of ER, PR, and HER2, constitutes 10–15% of all diagnoses and is frequently associated with deleterious mutations in breast cancer susceptibility gene 1 (*BRCA1*), a gene critical for DNA repair and genomic stability [5]. Among all subtypes, TNBC demonstrates the highest degree of clinical aggression, characterized by rapid progression, early relapse, and preferential metastasis to visceral organs, including the liver, lungs, and central nervous system [6]. The development of targeted therapies for TNBC has proven especially difficult owing to its pronounced molecular heterogeneity, and systemic treatment continues to rely predominantly on cytotoxic chemotherapy regimens [7]. Given that TNBC patients frequently demonstrate limited responses to immunotherapy and the absence of actionable molecular receptors precludes hormone-directed or HER2-targeted regimens, natural product-derived compounds have attracted renewed interest as multi-target agents with the potential to complement or overcome the limitations of existing treatments [8]. While early-stage disease may be addressed surgically, advanced stages are almost entirely dependent on chemotherapy, often with limited long-term efficacy [9]. The clinical utility of conventional treatments such as chemotherapy and radiotherapy is often hindered by severe systemic toxicity, emergence of multidrug resistance, and significant deterioration of patient quality of life [10]. Even with recent advances in targeted therapies and immune checkpoint inhibitors, treatment responses remain inconsistent, and toxicity profiles continue to pose significant challenges. These limitations underscore the urgent need to identify alternative therapeutic agents with higher efficacy and reduced systemic burden.

Natural products, a structurally diverse class of bioactive metabolites synthesized or accumulated by living organisms, have long served as a foundational resource in oncology drug discovery and development [11]. Across the history of therapeutic innovation, phytomedicines have remained integral to the clinical management of a broad spectrum of diseases [12]. Their translational success is exemplified by several landmark compounds. Paclitaxel, originally isolated from *Taxus brevifolia*, has become a cornerstone in the treatment of metastatic breast cancer [13], including in cases where resistance to other therapies has emerged. Berberine, derived from *Coptis chinensis* and other medicinal plants, continues to be widely employed against enteric pathogens such as *Shigella* and *Vibrio cholerae* [14]. Chloroquine, a synthetic 4-aminoquinoline antimalarial developed as an analog of quinine from *Cinchona* species, has historically served as a cornerstone of malaria therapy [15]. However, despite these successes, the transition of natural compounds from primary screening hits to clinically approved therapeutics remains constrained by challenges in pharmacokinetics, pharmacodynamics, and bioavailability. Among natural product classes, isoquinoline alkaloids—particularly those isolated from *Coptis chinensis* and *Stephania tetrandra*—have attracted considerable attention due to their polypharmacological activities and favorable safety profiles [11,16]. Characterized by isoquinoline or tetrahydroisoquinoline moieties, these compounds exert potent anti-cancer effects by modulating diverse oncogenic processes, including cell cycle arrest, apoptosis induction, angiogenesis inhibition, and suppression of epithelial–mesenchymal transition through the regulation of multiple intracellular signaling pathways [17,18,19].

**Figure 1:**
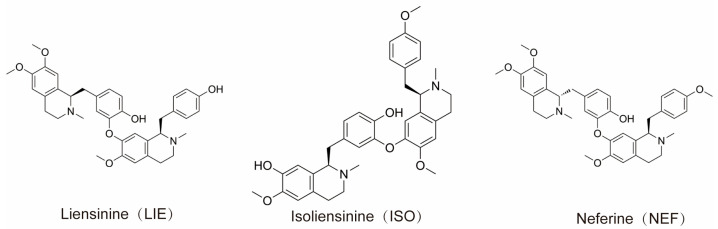
Chemical structures of the three principal bisbenzylisoquinoline alkaloids isolated from *Nelumbo nucifera* embryos: liensinine (LIE, C_37_H_42_N_2_O_6_), isoliensinine (ISO, C_37_H_42_N_2_O_6_), and neferine (NEF, C_38_H_44_N_2_O_6_). LIE and ISO are positional isomers differing in the position of the phenolic hydroxyl and methoxy substituents on the benzyl moieties, while NEF bears an additional N-methyl group relative to LIE. Structural formulas were obtained from MedChemExpress (MCE; www.medchemexpress.com).

Liensinine (LIE) and its structurally related monomeric analogs, isoliensinine (ISO) and neferine (NEF), constitute the principal bisbenzylisoquinoline alkaloids isolated from the embryos of *Nelumbo nucifera* (lotus seed hearts). LIE and ISO are positional isomers sharing the molecular formula C_37_H_42_N_2_O_6_, while NEF (C_38_H_44_N_2_O_6_) differs by containing an additional methyl group [20]. The chemical structures of LIE, ISO, and NEF are illustrated in Figure 1, highlighting the positional isomerism between LIE and ISO and the additional N-methyl substitution in NEF. Structural formulas were obtained from the compound datasheets provided by MedChemExpress (MCE; Monmouth Junction, NJ, USA; www.medchemexpress.com). A growing body of literature has documented the diverse pharmacological potential of these alkaloids, highlighting their significant anti-tumor, anti-inflammatory, and neuroprotective effects across multiple malignancies [11]. Preclinical studies have demonstrated robust anti-tumor activity of lotus-based compounds against breast, lung, and liver cancers. Notably, LIE and ISO have been shown to induce apoptosis in MDA-MB-231 and MCF-7 breast cancer cells through modulation of the BCL2-associated X protein (BAX)/B-cell lymphoma 2 (BCL-2) ratio and activation of the caspase-3/PARP signaling cascade. ISO has further been observed to selectively induce oxidative stress and activate the p38 MAPK/JNK pathway in TNBC cells, while sparing the normal human breast epithelial cell line MCF-10A [21]. Consistently, ISO has been reported to selectively trigger apoptosis in TNBC cells via reactive oxygen species (ROS) generation and p38 MAPK/JNK activation, with reduced cytotoxicity toward MCF-10A [22].

Despite these promising findings, comparative mechanistic insights into the anti-proliferative properties of LIE, ISO, and NEF remain limited. To address this gap, the present study evaluated the growth-inhibitory effects of these three alkaloids in HCC1954, MDA-MB-231, and MDA-MB-468 breast cancer cell lines using cell viability assays and flow cytometry. LIE, ISO, and NEF were selected as the focus compounds because: (1) they are the most abundant and commercially available (purity ≥ 99%) bisbenzylisoquinoline alkaloids in *Nelumbo nucifera* embryos; (2) LIE and ISO are positional isomers, and NEF differs by one additional methyl group, making this trio ideal for structure–activity relationship analysis; and (3) they represent the most extensively studied compounds within this alkaloid class. Transcriptomic profiling was employed to elucidate the molecular mechanisms underlying their anti-tumor activity. The primary objective of this study was to identify the most potent anti-proliferative agent among these structurally related bisbenzylisoquinoline alkaloids.

## 2. Results

### 2.1. LIE, ISO, and NEF Inhibit Breast Cancer Cell Proliferation in a Dose-Dependent Manner

To evaluate the anti-proliferative potential of LIE, ISO, and NEF, HCC1954, MDA-MB-468, and MDA-MB-231 cells were treated with increasing concentrations (3, 10, 30, 60, and 100 μM) of each compound for 48 h, followed by CCK-8 proliferation inhibition assays.

**Figure 2:**
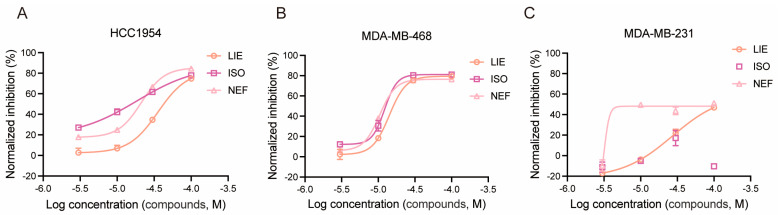
LIE, ISO, and NEF inhibit breast cancer cell proliferation in a dose-dependent manner. (**A**–**C**) Normalized proliferation inhibition (%) of HCC1954 (**A**), MDA-MB-468 (**B**), and MDA-MB-231 (**C**) cells treated with 0, 3, 10, 30, 60, or 100 μM LIE, ISO, or NEF for 48 h, as measured by CCK-8 assay. Dose–response curves were fitted by nonlinear regression. Data are expressed as mean ± SD from three independent experiments (*n* = 5).

All three alkaloids suppressed cell proliferation in a dose-dependent manner, though with cell line- and compound-specific potency profiles (Figure 2). In HCC1954 cells (Figure 2A), ISO and NEF exhibited markedly higher potency than LIE, as evidenced by their left-shifted dose–response curves. ISO and NEF achieved substantial inhibition of proliferation at concentrations as low as 3 µM, with IC_50_ values of approximately 18.52 µM and 21.26 µM, respectively, whereas LIE required considerably higher concentrations to achieve comparable inhibition, yielding an IC_50_ of approximately 35.76 µM. MDA-MB-468 cells were the most sensitive of the three lines to all three compounds (Figure 2B), with dose–response curves closely overlapping and IC_50_ values falling within a narrow range: LIE, ISO, and NEF inhibited proliferation with IC_50_ values of approximately 14.14, 12.19, and 10.46 µM, respectively. In contrast, MDA-MB-231 cells (Figure 2C) displayed a strikingly divergent response pattern. LIE and NEF produced clear sigmoidal dose–response curves with calculable IC_50_ values of approximately 27.22 and 31.82 µM, respectively. ISO, however, failed to elicit a consistent dose-dependent proliferation inhibition across the tested concentration range, with inhibition values remaining low and variable, precluding reliable IC_50_ determination in this cell line. This differential responsiveness, particularly the resistance of MDA-MB-231 to ISO, prompted further mechanistic investigation via transcriptomic profiling, cell cycle distribution, and apoptosis analysis. These findings corroborate earlier studies demonstrating dose-dependent growth inhibition of breast cancer cells by lotus-derived alkaloids [21].

### 2.2. LIE, NEF, and ISO Induce Robust Apoptosis in Breast Cancer Cells

**Figure 3:**
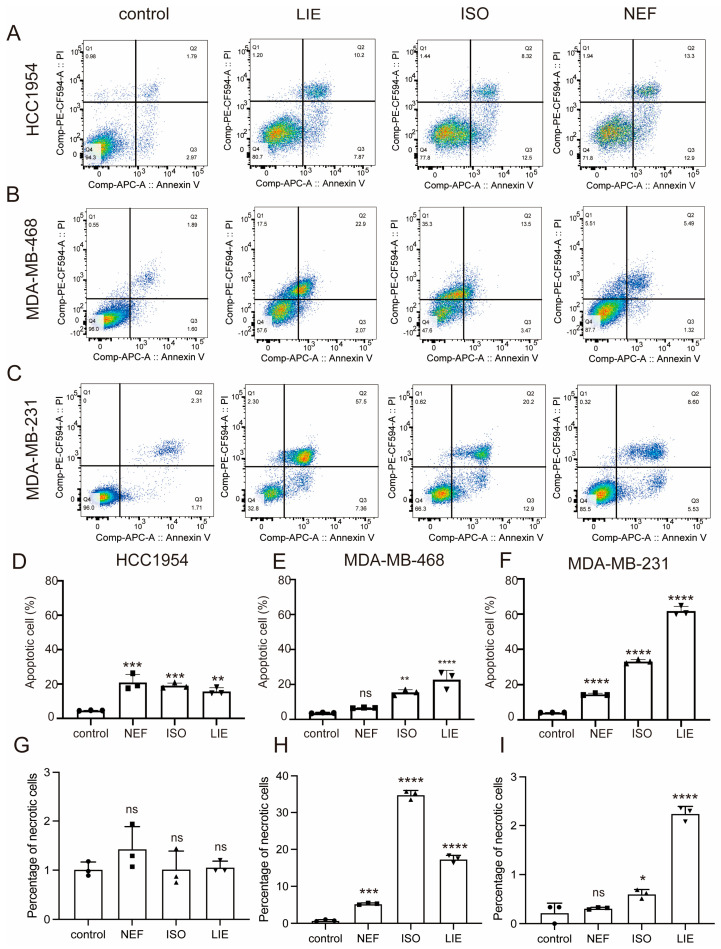
LIE, ISO, and NEF induce apoptosis in breast cancer cells. (**A**–**C**) Representative flow cytometry plots showing Annexin V-APC/PI staining in HCC1954 (**A**), MDA-MB-468 (**B**), and MDA-MB-231 (**C**) cells treated with 60 µM LIE, ISO, or NEF for 24 h. Quadrants indicate viable (Annexin V^−^, PI^−^; lower left), early apoptotic (Annexin V^+^, PI^−^; lower right), late apoptotic (Annexin V^+^, PI^+^; upper right) and necrotic (Annexin V^−^, PI^+^; upper left) populations. (**D**–**F**) Quantification of early and late apoptotic cells in HCC1954 (**D**), MDA-MB-468 (**E**), and MDA-MB-231 (**F**). Data are expressed as mean ± SD from three independent experiments (*n* = 3). Statistical significance was determined by one-way ANOVA and Dunnett’s post hoc test: * *p* < 0.05, ** *p* < 0.01, *** *p* < 0.001, **** *p* < 0.0001 vs. control; ns = not significant. (**G**–**I**) Quantification of necrotic (Annexin V^−^/PI^+^) populations in HCC1954 (**G**), MDA-MB-468 (**H**), and MDA-MB-231 (**I**) cells, confirming that none of the compounds significantly increased necrosis relative to control.

To further explore the pro-apoptotic potential of the three compounds, Annexin V-APC/PI double-staining was performed in HCC1954, MDA-MB-468, and MDA-MB-231 cell lines following treatment with 60 μM of LIE, ISO, or NEF. Flow cytometric analysis revealed that all three compounds triggered apoptosis to varying degrees across the tested models. In HCC1954 cells, treatment with LIE, ISO, or NEF significantly increased apoptotic rates relative to untreated controls, increasing from 4.76% to 15–20% (*p* < 0.001, Figure 3A–C). MDA-MB-468 cells exhibited strong apoptotic responses to LIE and ISO (*p* < 0.0001 and *p* < 0.01, respectively), whereas NEF had a negligible pro-apoptotic effect (*p* > 0.05, Figure 3E). The most pronounced induction of apoptosis was observed in MDA-MB-231 cells (Figure 3C,F), where LIE markedly reduced viable cells from 96.0% to 32.8%, while late apoptotic cells increased significantly from 2.31% to 57.5%. ISO and NEF also significantly elevated apoptosis in MDA-MB-231 cells, with apoptotic populations reaching 33.1% and 14.1%, respectively (*p* < 0.0001, Figure 3F). The results collectively demonstrate that LIE, ISO, and NEF exert potent and statistically significant pro-apoptotic effects in breast cancer cells, with cell line-specific sensitivity profiles. To distinguish apoptotic from necrotic cell death, the percentages of necrotic cells (Annexin V^−^/PI^+^, Q1) were also quantified across all treatment conditions (Figure 3G–I). In HCC1954 cells (Figure 3G), necrotic populations remained consistently low across all three treatments (NEF: ~1.5%; ISO: ~1.0%; LIE: ~1.0%), comparable to the vehicle control (~1.0%), indicating that the observed cell death in this line was attributable predominantly to apoptosis rather than necrosis. Similarly, in MDA-MB-231 cells (Figure 3I), necrotic fractions were minimal across all conditions (NEF: ~0.4%; ISO: ~0.6%; LIE: ~2.3%), confirming that the substantial apoptotic induction observed with LIE and ISO in this cell line occurred without concurrent necrotic cell death. In contrast, MDA-MB-468 cells (Figure 3H) displayed notably elevated necrotic populations following ISO (~35%) and LIE (~17%) treatment, which were substantially higher than the vehicle control (~1%) and higher than necrotic fractions observed in the other two cell lines. This finding suggests that LIE and ISO may engage additional cell death mechanisms in MDA-MB-468 cells beyond canonical apoptosis. This elevated Q1 population in MDA-MB-468 cells is most likely attributable to secondary necrosis arising from late-stage apoptosis rather than primary necrotic cell death, given the particularly robust apoptotic response observed in this cell line. It should be noted that standard Annexin V/PI flow cytometry cannot distinguish between primary necrosis and secondary necrosis based on staining pattern alone, and this represents an inherent limitation of the assay. Collectively, these data demonstrate that while apoptosis is the predominant mode of cell death induced by all three compounds across the three breast cancer subtypes tested, the relative contribution of necrosis is cell line- and compound-dependent, with MDA-MB-468 cells showing a distinctly higher susceptibility to ISO- and LIE-induced necrotic death.

### 2.3. Cell Cycle Disruption by LIE, ISO, and NEF in Breast Cancer Cells

**Figure 4:**
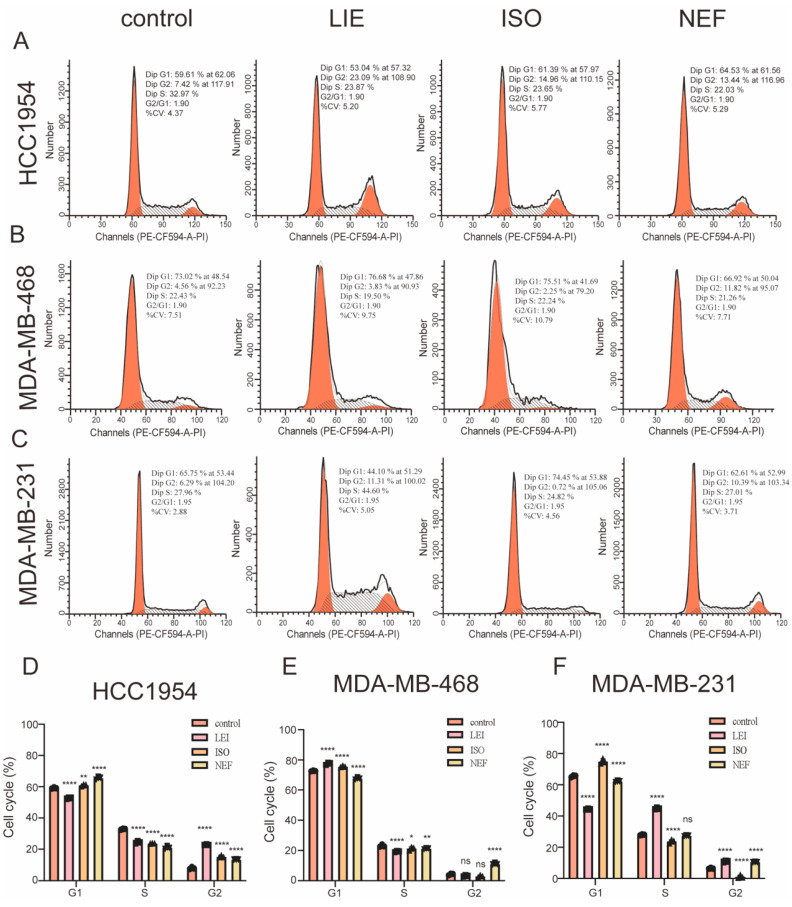
LIE, ISO, and NEF induce cell cycle arrest in breast cancer cells. (**A**–**C**) Representative DNA content histograms obtained by flow cytometry for HCC1954 (**A**), MDA-MB-468 (**B**), and MDA-MB-231 (**C**) cells following treatment with 60 µM LIE, ISO, or NEF for 24 h. Orange peaks denote G1 and G2/M phases, while hatched areas represent S phase. (**D**–**F**) Quantitative distribution of cells in G1, S, and G2 phases for HCC1954 (**D**), MDA-MB-468 (**E**), and MDA-MB-231 (**F**). Data are expressed as mean ± SD from three independent biological replicates (*n* = 3). Statistical significance was determined using two-way ANOVA followed by Dunnett’s multiple comparisons test: * *p* < 0.05, ** *p* < 0.01, *** *p* < 0.001, **** *p* < 0.0001 vs. control; ns, not significant.

To assess whether the anti-proliferative activity of LIE, ISO, and NEF involves disruption of cell cycle progression, cell cycle profiles were analyzed in HCC1954, MDA-MB-468, and MDA-MB-231 breast cancer cell lines after 24 h of treatment with 60 µM of each compound. Flow cytometry revealed compound- and cell line-specific alterations in cell cycle distribution (Figure 4A–C). In HCC1954 cells (Figure 4A), treatment with LIE, ISO, or NEF resulted in a marked accumulation of cells in the G2 phase, indicating effective blockade of cell cycle progression prior to mitosis. In MDA-MB-468 cells (Figure 4B), LIE induced a pronounced G2-phase arrest, increasing the G2 population from 4.56% in control cells to 11.82% (*p* < 0.0001). This was accompanied by a significant increase in the S-phase fraction, further supporting impaired cell cycle progression following alkaloid exposure. In MDA-MB-231 cells (Figure 4C), LIE similarly triggered robust G2 arrest, with the G2 population rising from 6.29% in controls to 23.87% (*p* < 0.0001). In contrast, ISO treatment in this cell line predominantly induced G1-phase arrest (74.45% vs. 65.75% in controls), whereas NEF primarily promoted G2-phase accumulation. Although the specific phase of arrest differed among compounds and cell lines, a consistent pattern emerged in which all three bisbenzylisoquinoline alkaloids disrupted normal cell cycle progression through arrest at the G1 and/or G2 phases. This enforced blockade of cell cycle transition to mitosis provides a mechanistic basis for the suppression of proliferative capacity observed in the CCK-8 viability assays.

### 2.4. Transcriptomic Profiling Reveals Distinct Gene Expression Programs and Signaling Pathway Perturbations Induced by LIE, ISO, and NEF

**Figure 5:**
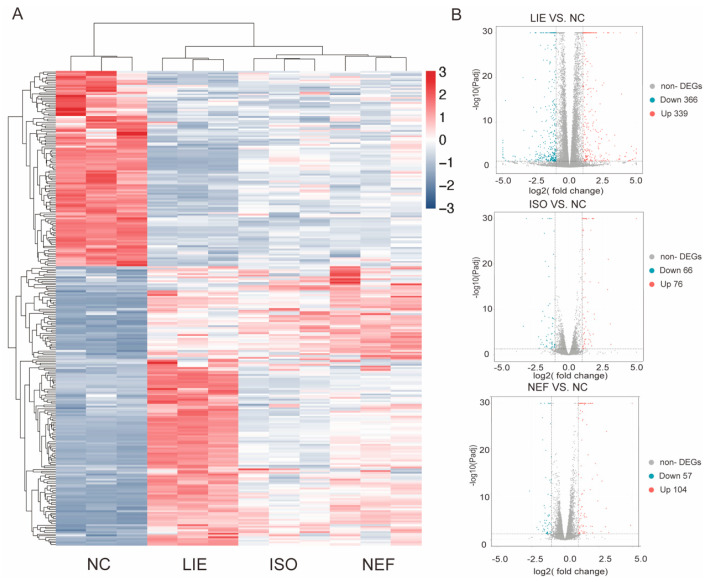
Transcriptomic profiling of HCC1954 cells treated with LIE, ISO, or NEF. (**A**) Hierarchical clustering of DEGs following 24 h exposure to each compound (30 µM). Color scale represents Z-score-normalized expression (red: up-regulation; blue: down-regulation). (**B**) Volcano plots showing DEGs in each treatment group relative to the control. Red dots: up-regulated genes; blue dots: down-regulated genes; gray dots: non-significant genes. Thresholds: |log2(fold change)| ≥ 1 and adjusted *p* ≤ 0.05.

To elucidate the molecular mechanisms underlying the anti-cancer activities of LIE, ISO, and NEF, transcriptomic profiling was performed in HCC1954 cells following 24 h exposure to each compound (30 µM). HCC1954 cells were selected for their consistent and intermediate sensitivity to all three alkaloids, providing a balanced model for comparative analysis of transcriptional responses while minimizing cell type-specific resistance artifacts. Hierarchical clustering of DEGs revealed distinct compound-specific transcriptional signatures (Figure 5A). Among the three treatments, LIE induced the most extensive transcriptional reprogramming, with its expression profile clearly separated from the control group and from those of ISO and NEF. ISO and NEF treatments produced comparatively moderate and overlapping transcriptional responses, while LIE elicited a broader and distinct gene expression shift, indicative of unique regulatory activity. Volcano plot analysis further quantified the scale of transcriptional perturbation (Figure 5B). LIE treatment resulted in 705 DEGs (339 up-regulated and 366 down-regulated), substantially exceeding the number identified in ISO (142 DEGs: 76 up-regulated and 66 down-regulated) and NEF (161 DEGs: 104 up-regulated and 57 down-regulated). The magnitude and breadth of LIE-induced transcriptional alterations suggest more pervasive engagement of regulatory networks, aligning with its potent anti-proliferative phenotype observed in cell viability assays. These compound-specific DEG profiles offer a molecular basis for the divergent effects of LIE, ISO, and NEF on breast cancer cell growth.

**Figure 6:**
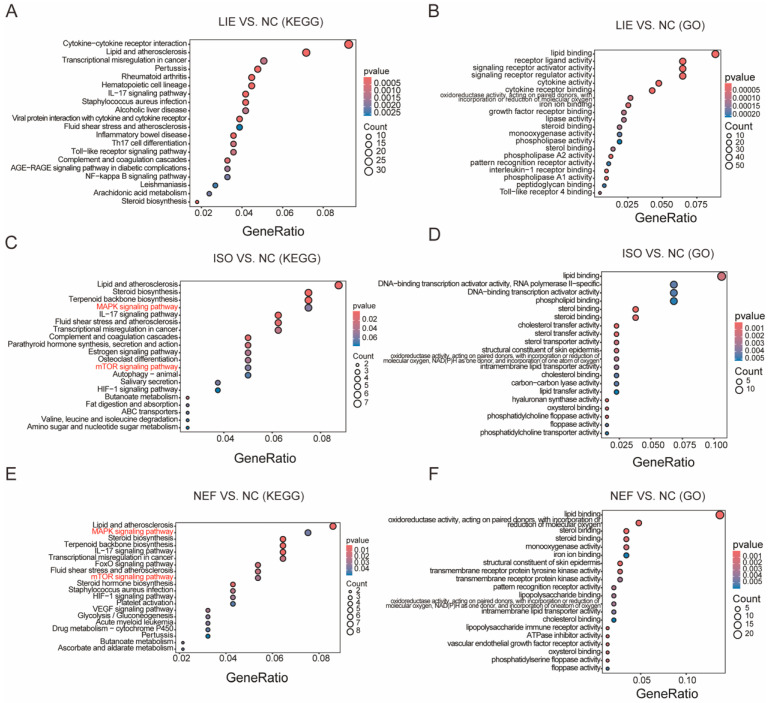
Functional enrichment analysis of DEGs in HCC1954 cells treated with LIE, ISO, or NEF. (**A**,**C**,**E**) KEGG pathway enrichment bubble plots for LIE (**A**), ISO (**C**), and NEF (**E**) compared to control. Bubble size denotes the number of genes enriched per pathway, and bubble color reflects statistical significance (*p*-value). (**B**,**D**,**F**) Gene Ontology (GO) molecular function (MF) enrichment bar charts for LIE (**B**), ISO (**D**), and NEF (**F**). Bar color intensity represents *p*-value significance.

To further dissect the functional relevance of these transcriptional alterations, KEGG pathway and GO functional enrichment analyses were conducted (Figure 6). KEGG enrichment revealed both shared and compound-specific pathways (Figure 6A,C,E). All three alkaloids significantly enriched the “Lipid and atherosclerosis” pathway, implicating conserved effects on lipid-associated signaling in their anti-cancer activities. However, each compound also demonstrated unique pathway enrichment profiles. LIE exhibited broader pathway perturbations, with notable enrichment in inflammation-related pathways, including “Cytokine-cytokine receptor interaction” and “Viral protein interaction with cytokine and cytokine receptor”; immune-related pathways, including “Pertussis”, “Rheumatoid arthritis”, and “Inflammatory bowel disease”; and critical cancer-related pathways, including “Toll-like receptor signaling pathway”, “NF-kappa B signaling pathway”, and “AGE-RAGE signaling pathway in diabetic complications”. In contrast, ISO and NEF predominantly enriched signaling and metabolic pathways directly implicated in oncogenesis (Figure 6C,E). Both compounds significantly perturbed the “MAPK signaling pathway” and “mTOR signaling pathway”, key regulators of cell proliferation, growth, and survival, and notably implicated in breast cancer progression and therapeutic resistance. NEF also enriched several biosynthetic and metabolic pathways, including “Steroid biosynthesis”, “Terpenoid backbone biosynthesis”, and “Drug metabolism”, indicating broader metabolic engagement.

GO molecular function analysis further delineated the biological activities encoded by the DEGs (Figure 6B,D,F). Across all three treatments, analysis revealed enrichment of lipid-related terms, including “lipid binding”, “receptor ligand activity”, and various oxidoreductase functions, reinforcing KEGG findings. LIE was uniquely associated with enrichment in signaling receptor-related functions, including “signaling receptor regulator activity”, “cytokine activity”, and “growth factor receptor binding”, consistent with its strong activation of cytokine and immune-related pathways. ISO and NEF enriched DNA-binding transcription factor activities, steroid-related functions, cholesterol transfer and transport activities, and enzymatic activities involved in metabolic processes. Collectively, these transcriptomic and pathway analyses reveal that LIE, ISO, and NEF exert their anti-cancer effects through distinct but partially overlapping mechanisms. ISO and NEF converge on inhibition of MAPK and mTOR signaling pathways, which are central to cancer cell survival and therapeutic resistance. LIE additionally modulates immune and inflammatory signaling, suggesting a broader mechanism that may involve tumor–microenvironment crosstalk. These insights provide a mechanistic framework linking transcriptional regulation to the induction of apoptosis and cell cycle arrest observed across the alkaloid-treated breast cancer models.

### 2.5. Protein-Level Validation Confirms Inhibition of MAPK and AKT Signaling Pathways by LIE, ISO, and NEF

Transcriptomic analysis identified significant enrichment of the MAPK and mTOR signaling pathways following treatment with LIE, ISO, and NEF, implicating these pathways as central regulators of the anti-cancer activity of the three compounds. Given the established role of MAPK and AKT/mTOR cascades in controlling proliferation, survival, and apoptosis—and their frequent dysregulation in breast cancer [23,24]—Western blot analysis was performed to validate these transcriptomic findings at the protein level.

**Figure 7:**
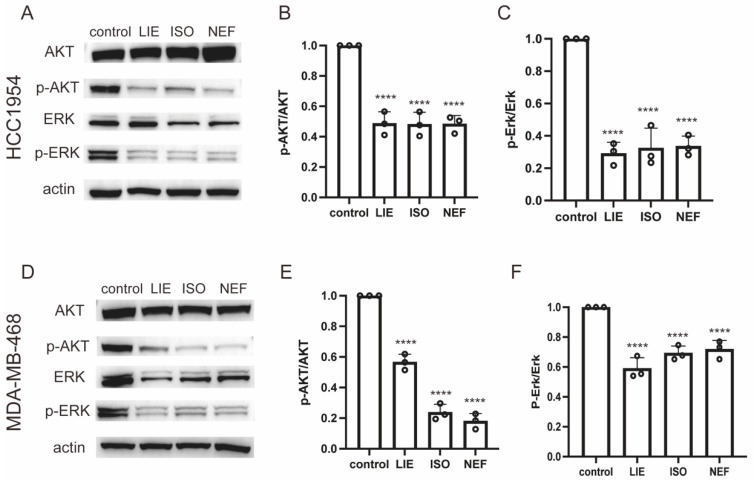
LIE, ISO, and NEF inhibit AKT and ERK phosphorylation in breast cancer cells. (**A**) Western blot analysis of AKT, p-AKT, ERK, and p-ERK in HCC1954 cells treated with 30 µM LIE, ISO, or NEF for 24 h. Actin served as a loading control. (**B**,**C**) Densitometric quantification of p-AKT/AKT (**B**) and p-ERK/ERK (**C**) ratios in HCC1954 cells. (**D**) Western blot analysis of AKT, p-AKT, ERK, and p-ERK protein levels in MDA-MB-468 cells treated with 30 µM LIE, ISO, or NEF for 24 h. Actin served as a loading control. (**E**,**F**) Densitometric quantification of p-AKT/AKT (**E**) and p-ERK/ERK (**F**) ratios in MDA-MB-468 cells. Data are expressed as mean ± SD from three independent experiments (*n* = 3). **** *p* < 0.0001 vs. control.

HCC1954 and MDA-MB-468 cells were treated with 30 µM LIE, ISO, or NEF for 24 h, and the phosphorylation status of ERK and AKT was assessed. In HCC1954 cells (Figure 7A), all three compounds markedly suppressed phosphorylation of both AKT (p-AKT) and ERK (p-ERK), with no detectable changes in total protein levels. Densitometric quantification revealed that LIE, ISO, and NEF reduced p-AKT levels to approximately 50%, 47%, and 46% of the control, respectively (*p* < 0.0001 for all treatments; Figure 7B). Similarly, p-ERK levels were significantly decreased to 32%, 32%, and 33% of control levels, respectively (*p* < 0.0001 for all treatments; Figure 7C). These results confirm that all three alkaloids effectively inhibit AKT and MAPK/ERK signaling in HCC1954 cells with comparable potency.

Consistent effects were observed in MDA-MB-468 cells (Figure 7D). LIE, ISO, and NEF treatment reduced p-AKT levels to 56%, 23%, and 18% of control levels, respectively (*p* < 0.0001; Figure 7E), indicating stronger AKT suppression by ISO and NEF. All three compounds also decreased p-ERK levels to 58%, 69%, and 71% of control levels, respectively (*p* < 0.0001; Figure 7F). Interestingly, while LIE demonstrated slightly stronger ERK inhibition in this cell line, all three compounds exhibited significant inhibitory activity.

Collectively, these results provide direct protein-level validation of the transcriptomic data, demonstrating that these alkaloids exert their anti-cancer effects through suppression of the MAPK/ERK and AKT signaling pathways. The observed downregulation of p-AKT and p-ERK aligns with the apoptosis induction and cell cycle arrest observed in earlier assays. Moreover, the variation in inhibition profiles between HCC1954 and MDA-MB-468 cells suggests that the specific molecular context of each cell line may influence the relative potency of these compounds against individual pathway components. Nevertheless, the consistent suppression of both AKT and ERK phosphorylation across models underscores the broad therapeutic potential of these natural lotus-derived alkaloids and validates the pathway-level regulatory activity inferred from transcriptomic analysis.

## 3. Discussion

TNBC, representing approximately 10–15% of breast cancer cases, is characterized by aggressive clinical behavior, poor prognosis, and high rates of metastasis and recurrence [25]. The absence of well-defined molecular targets (ER, PR, and HER2) precludes the use of directed therapies routinely employed in other breast cancer subtypes. Consequently, therapeutic strategies for TNBC remain largely limited to cytotoxic chemotherapy, to which fewer than 20% of patients respond favorably. As such, most patients ultimately develop chemoresistance and experience early relapse [26,27]. This therapeutic inadequacy underscores the urgent need for multi-target bioactive agents capable of circumventing adaptive resistance. LIE and its structural analogs (ISO and NEF) represent promising candidates in this regard [28]. The present study demonstrated that these three alkaloids exert potent inhibitory effects on two central oncogenic cascades in TNBC: the MAPK and mTOR signaling pathways. Targeting both pathways simultaneously confers a mechanistic advantage over conventional monotherapies that often fail due to feedback activation and pathway compensation. Intriguing, despite their structural similarity, each compound exhibited distinct regulatory activity, implicating divergent structure–activity relationships as determinants of functional specificity.

To delineate these differences, transcriptomic profiling was conducted in HCC1954 cells. RNA-seq analysis demonstrated that LIE induced substantially broader transcriptional reprogramming, with 705 DEGs compared to 142 for ISO and 161 for NEF, suggesting a structurally optimized scaffold that enables more comprehensive regulatory engagement within the cellular transcriptome. This enhanced activity is likely attributable to the stereochemical configuration and substitution pattern of the phenolic hydroxyl and methoxy groups, which may facilitate improved cellular permeability and more effective engagement of core regulatory proteins in breast cancer cells [29]. Prior studies have identified methylation status and spatial arrangement of bisbenzylisoquinoline phenolic ring structures as key modulators of membrane permeability and target protein binding.

Collectively, this study revealed a profound transcriptional reprogramming that resulted in multiple phenotypic outcomes, primarily characterized by the induction of programmed cell death and the suppression of cell cycle progression. The marked increase in somatic tumor cell death across diverse breast cancer cell lines following LIE, ISO, and NEF exposure is attributable to activation of the late apoptotic cascade, consistent with irreversible cellular demise rather than cytostatic arrest. This cytotoxicity is mechanistically linked to a pronounced G2/M-phase blockade at the cell cycle checkpoint, a critical barrier preventing mitotic entry of genetically unstable cells [30], thereby limiting aberrant proliferation—a hallmark of malignant progression [31]. Enrichment of the MAPK and mTOR signaling pathways in transcriptomic analyses provided additional molecular support for these effects. Simultaneous disruption of these core oncogenic pathways imposes convergent stress sufficient to drive cells out of the proliferative cycle and into apoptotic execution [32]. This dual-pathway inhibition highlights a promising multi-modal strategy, wherein lotus-derived alkaloids may circumvent compensatory survival mechanisms that frequently underlie resistance to monotherapies.

While this study offers mechanistic insight into the anti-tumor activities of these alkaloids, several limitations remain. Most critically, the primary molecular targets through which LIE, ISO, and NEF exert their effects have yet to be fully delineated; rather than lacking defined molecular targets, LIE and its analogs are characterized by broad, multitarget engagement. Published studies have identified G-quadruplex DNA structures [33], CDK2/CDK4 [34], the protein tyrosine phosphatase SHP2 [35], and the voltage-gated potassium channel Kv10.1 [36] as relevant targets. This polypharmacology may contribute to the breadth of anti-cancer effects but complicates mechanistic interpretation and limits rational structure–activity optimization [14]. Published studies also support the drug discovery potential of these compounds: they exhibit moderate oral bioavailability with hepatic CYP450-mediated demethylation, and molecular docking and ADMET analyses indicate acceptable Lipinski compliance, moderate membrane permeability, and engagement with kinase domains consistent with MAPK/AKT suppression. Computationally predicted targets include CDK2, CDK4, ERK1/2, and AKT. Structure–activity relationship (SAR) studies have examined semi-synthetic analogs; differences in methyl substitution and hydroxyl positioning have been identified as key activity modulators, though moderate potency and multitarget polypharmacology remain limitations requiring rational analog design. Regarding stability, these compounds are dissolved in DMSO and stored at −20 °C (chemically stable for >12 months). Published pharmacokinetic data suggest moderate hepatic first-pass metabolism via CYP3A4-mediated demethylation; formulation strategies such as nanoparticle encapsulation may improve systemic bioavailability in future in vivo studies [37]. Additionally, affinity-based proteomics platforms—such as drug affinity chromatography and thermal proteome profiling (TPP)—are currently being applied to systematically identify direct binding partners [38]. Additionally, although robust cytotoxic activity was demonstrated in vitro, validation in in vivo models remains essential. Preclinical testing in xenograft models is required to evaluate how tumor microenvironmental complexity influences compound bioavailability, distribution, and efficacy [39]. Finally, substantial variation in pharmacokinetic and pharmacodynamic properties across these alkaloids underscores the need for rigorous PK/PD profiling to optimize dosing strategies and ensure systemic stability, effective bioactivity, and safe metabolic clearance [40].

Planned future research will transition toward in vivo evaluation of the anti-tumor efficacy and toxicity of LIE, ISO, and NEF in murine models, aiming to establish translational relevance for this class of lotus-derived compounds. This phase is critical to bridging the preclinical-to-clinical divide, in alignment with the developmental trajectories of other validated anti-cancer agents [39]. Functional investigation of MAPK and mTOR pathway components is also a priority, employing CRISPR/Cas9-mediated gene editing or RNA interference to confirm the causal role of key effectors in mediating the phenotypic outcomes observed in vitro [41,42]. Additionally, efforts are underway to formulate synergistic treatment strategies by combining these natural compounds with frontline chemotherapeutics, such as paclitaxel and cisplatin. Previous studies have suggested that such combinations may enhance therapeutic sensitivity [43,44] while mitigating dose-dependent toxicities commonly associated with monotherapy regimens [45].

## 4. Materials and Methods

### 4.1. Compounds

LIE (purity ≥ 99%; CAS: 2586-96-1), ISO (purity ≥ 99%; CAS: 6817-41-0), and NEF (purity ≥ 99%; CAS: 2292-16-2) were purchased from MedChemExpress (MCE, Monmouth Junction, NJ, USA; Cat# HY-N0484, HY-N0770, and HY-N0441, respectively). All compounds were dissolved in dimethyl sulfoxide (DMSO) to prepare stock solutions and stored at −20 °C.

### 4.2. Breast Cancer Cell Cultures

Human breast cancer cell lines MDA-MB-231, MDA-MB-468, and HCC1954 were obtained from Procell Corporation (CL-0150B, CL-0290B and CL-0756) and authenticated by short tandem repeat (STR) profiling. These cell lines correspond to the following ATCC reference strains (ATCC, Manassas, VA, USA): MDA-MB-231 (ATCC^®^ HTB-26™; triple-negative, ER^−^/PR^−^/HER2^−^), MDA-MB-468 (ATCC^®^ HTB-132™; triple-negative, ER^−^/PR^−^/HER2^−^, EGFR-overexpressing), and HCC1954 (ATCC^®^ CRL-2338™; HER2-positive, ER^−^/PR^−^/HER2^+^). MDA-MB-231 and MDA-MB-468 cells were cultured in Dulbecco’s Modified Eagle Medium (DMEM, Thermo Fisher Scientific, Waltham, MA, USA), while HCC1954 cells were cultured in RPMI-1640 medium (Thermo Fisher Scientific). All media were supplemented with 10% fetal bovine serum (FBS) and 1% penicillin–streptomycin. Cells were maintained at 37 °C in a humidified incubator with 5% CO_2_.

### 4.3. CCK-8 Assay

The effects of LIE, ISO, and NEF on cell proliferation inhibition were evaluated using the Cell Counting Kit-8 (CCK-8; ACE Biotechnology, Zhuzhou, China). Briefly, MDA-MB-231, MDA-MB-468, and HCC1954 cells were seeded in 96-well plates at a density of 3000 cells per well and allowed to adhere overnight. Cells were then exposed to LIE, ISO, and NEF at concentrations of 0, 3, 10, 30, 60, and 100 µM for 48 h. The 48 h incubation time was selected based on pilot experiments, in which 24 h treatment showed insufficient and highly variable inhibition; 48 h captures at least one full cell division cycle and enables more reproducible dose–response measurements. Following treatment, the medium was replaced with fresh medium containing 10% (*v*/*v*) CCK-8 reagent and incubated for 30 min at 37 °C. Absorbance at 450 nm was measured using a microplate SpectraMax Absorbance Reader (CMax Plus, Molecular Devices, San Jose, CA, USA). All experiments were performed in triplicate, and proliferation inhibition was expressed as a percentage relative to untreated controls.

### 4.4. Apoptosis Assay (Annexin V/Propidium Iodide (PI) Staining)

Apoptosis was quantified using an Annexin V-FITC/PI Apoptosis Detection Kit (HY-K1073, MedChemExpress, Monmouth Junction, NJ, USA). Briefly, MDA-MB-231, MDA-MB-468, and HCC1954 cells were seeded in 6-well plates at a density of 2 × 10^5^ cells per well and treated with 60 µM LIE, ISO, or NEF for 24 h. After treatment, cells were harvested, washed with cold phosphate-buffered saline (PBS), and stained with Annexin V-FITC and PI according to the manufacturer’s instructions. Stained cells were analyzed by flow cytometry (BD FACSCanto II, Franklin Lakes, NJ, USA). T APC fluorescence was detected using a 633 nm red laser with a 660/20 nm bandpass filter; PI fluorescence was detected using a 488 nm blue laser with a 616/23 nm bandpass filter. The percentage of Annexin V-positive cells was used to determine apoptosis levels.

### 4.5. Cell Cycle Analysis

Cell cycle distribution was determined by PI staining and flow cytometry. Briefly, MDA-MB-231, MDA-MB-468, and HCC1954 cells were seeded in 6-well plates at a density of 2 × 10^5^ cells per well and treated with 60 µM LIE, ISO, or NEF for 24 h. Following treatment, cells were harvested, washed with cold PBS, and fixed in 70% ethanol at 4 °C overnight. Cells were then treated with RNase A, stained with PI, and analyzed by flow cytometry (BD FACSCanto II, USA). The percentages of cells in the G0/G1, S, and G2/M phases were determined.

### 4.6. RNA Sequencing (RNA-Seq)

Total RNA was extracted from HCC1954 cells treated with 30 µM LIE, ISO, or NEF for 24 h using TRIzol reagent (Invitrogen, Carlsbad, CA, USA), following the manufacturer’s protocols. The 30 µM concentration was selected as a sub-maximal dose that preserves RNA integrity; 60 µM causes extensive cell death, compromising RNA integrity number (RIN) scores and total RNA yield. RNA concentration, purity, and integrity were assessed using a spectrophotometer (Thermo Fisher Scientific, Wilmington, DE, USA) and an Agilent 2100 Bioanalyzer (Santa Clara, CA, USA). mRNA was enriched with oligo(dT) magnetic beads and fragmented prior to reverse transcription into cDNA. Sequencing libraries were constructed using the MGIEasy Universal Library Conversion Kit (APP-A, Shenzhen, China), purified with AMPure XP beads, and quantified with a Qubit 4.0 Fluorometer (Thermo Fisher Scientific). Paired-end sequencing (150 bp) was performed on the DNBSEQ-T7 platform. Differentially expressed genes (DEGs) were identified using DESeq2 (version 1.44.0) with thresholds of |fold change| ≥ 1 and *p* ≤ 0.05. Functional enrichment analysis of DEGs was performed based on Gene Ontology (GO) terms and Kyoto Encyclopedia of Genes and Genomes (KEGG) pathways.

### 4.7. Western Blot Analysis

To examine the effects of the three alkaloids on key signaling pathways, MDA-MB-231, MDA-MB-468, and HCC1954 cells were treated with 30 µM LIE, ISO, or NEF for 24 h. This sub-maximal concentration was chosen to capture early signaling changes while preserving sufficient protein yield; 60 µM causes extensive cell death that would compromise protein quantity and quality. Cells were then harvested and lysed to extract total proteins. Protein concentrations were determined using a BCA assay. Equal amounts of protein were separated by sodium dodecyl sulfate–polyacrylamide gel electrophoresis (SDS-PAGE) and subsequently transferred onto nitrocellulose membranes. Membranes were blocked and incubated overnight at 4 °C with primary antibodies against actin, p-ERK, ERK, AKT, and p-AKT, followed by washing and incubation with appropriate horseradish peroxidase (HRP)-conjugated secondary antibodies. Protein bands were visualized using an enhanced chemiluminescence (ECL) substrate and detected with the Tanon 5200 chemiluminescence imaging system (Shanghai, China). Actin was used as a loading control.

### 4.8. Statistical Analysis

All experimental results were derived from at least three independent biological replicates, expressed as mean ± standard deviation (SD). Statistical analysis was carried out using GraphPad Prism software (v9.0). For comparisons between two groups, unpaired Student’s *t*-tests were applied. For comparisons among more than two groups, one-way analysis of variance (ANOVA) followed by Dunnett’s multiple comparison test was used to determine significance. Two-way ANOVA was applied for experiments involving two factors (e.g., treatment and time or concentration), such as cell cycle analysis. Statistical significance was defined as *p* < 0.05 (* *p* < 0.05, ** *p* < 0.01, *** *p* < 0.001, **** *p* < 0.0001). For RNA-seq data, DEGs were identified using the DESeq2 algorithm with a |fold change| ≥ 1 and adjusted *p* (Padj) ≤ 0.05.

## 5. Conclusions

In conclusion, this study established LIE and structurally analogous bisbenzylisoquinoline alkaloids as compelling candidates for breast cancer intervention. Integration of high-throughput transcriptomic profiling with functional assays across multiple in vitro breast cancer models demonstrated potent induction of apoptosis and cell cycle arrest, underpinned by widespread transcriptional disruption [46]. These results provide compelling preclinical rationale for further exploration of the therapeutic potential of *N. nucifera*-derived compounds. Subsequent studies should delineate direct molecular targets and validate anti-tumor activity under physiological conditions to fully define the scope of application across breast cancer subtypes.

## Data Availability

The original data presented in the study are openly available in Zenodo at https://zenodo.org/records/18620430 (accessed on 11 February 2026).
